# A prospective randomized multicenter phase I/II clinical trial to evaluate safety and efficacy of NOVOCART disk plus autologous disk chondrocyte transplantation in the treatment of nucleotomized and degenerative lumbar disks to avoid secondary disease: safety results of Phase I—a short report

**DOI:** 10.1007/s10143-016-0781-0

**Published:** 2016-08-27

**Authors:** Anja Tschugg, Michael Diepers, Steinert Simone, Felix Michnacs, Sebastian Quirbach, Martin Strowitzki, Hans Jörg Meisel, Claudius Thomé

**Affiliations:** 1 Department of Neurosurgery, Medical University of Innsbruck, Anichstr. 35, A-6020 Innsbruck, Austria; 2 Department of Neuroradiology, Cantonal Hospital Aarau, Aarau, Switzerland; 3 TETEC Tissue Engineering Technologies AG, Reutlingen, Germany; 4 Neurocenter, Trauma Center Murnau, Murnau, Germany; 5 Department of Neurosurgery, BG-Clinic Bergmannstrost, Halle, Germany

**Keywords:** Autologous disk chondrocyte transplantation, Degenerative disk disease, Lumbar back pain, Sequestrectomy

## Abstract

NOVOCART^®^ Disk plus, an autologous cell compound for autologous disk chondrocyte transplantation, was developed to reduce the degenerative sequel after lumbar disk surgery or to prophylactically avoid degeneration in adjacent disks, if present. The NDisc trial is an ongoing multi-center, randomized study with a sequential phase I study within the combined phase I/II trial with close monitoring of tolerability and safety. Twenty-four adult patients were randomized and treated with the investigational medicinal product NDisc plus or the carrier material only. Rates of adverse events in Phase I of this trial were comparable with those expected in the early time course after elective disk surgery. There was one reherniation 7 months after transplantation, which corresponds to an expected reherniation rate. Immunological markers like CRP and IL-6 were not significantly elevated and there were no imaging abnormalities. No indications of harmful material extrusion or immunological consequences due to the investigational medicinal product NDplus were observed. Therefore, the study appears to be safe and feasible. Safety analyses of Phase I of this trial indicate a relatively low risk considering the benefits that patients with debilitating degenerative disk disease may gain.

## Introduction

Intervertebral disk degeneration (IVDD) is emphasized as an important cause of low back pain (LBP) [1]. Current treatment strategies are aggressive removal of the pathological disk or spondylodesis that do not address options of restoring structural or biological deteriorations of the intervertebral disk (IVD) as the underlying problem. Therefore, disk repair procedures are of interest to spine surgeons, because they offer a less invasive and biological alternative to lumbar fusion in an attempt to obviate LBP associated with IVDD earlier in the degenerative cascade. Advances in molecular biology are now encouraging innovative biological repair strategies. Different treatment modalities in vivo and in vitro include the administration of growth factors, the application of autologous or allogenic cells, gene therapeutic approaches, in situ therapy and the introduction of biomaterials, or a combination thereof [2–7]. Especially, reinsertion of autologous nucleus pulpous cells or stem cells delays degeneration in experimental in vivo or in vitro models of disk degeneration [8–10]. In humans, the IVD potentially be repaired after a herniation by transplanting the patient’s disk chondrocyte. According to this method, which is called the autologous disk-derived chondrocyte transplantation (ADCT), the disk tissue which herniated through the annulus fibrosus is removed at an initial surgical intervention. The chondrocytes are then enzymatically isolated, expanded in vitro and the suspension is reinjected into the damaged IVD [11].

Transplanted cultivated cells have to survive in a harsh environment in the disk space with low nutrients and a high amount of pro-inflammatory cytokines [12]. Therefore, NOVOCART^®^ Disk plus (NDplus) consists of a hydrogel with anti-inflammatory, anti-angiogenic, anti-osteogenic properties, and autologous disk cells for ADCT. This biomaterial is able to achieve a positive milieu conditioning of the previously damaged intervertebral disk, and thus, is intended to increase the survival rate and regeneration capability of the transplanted cells. Furthermore, the liquid biomaterial polymerizes after being injected, which should improve the cells’adherence.

NDplus was developed to provide rehydration and biological integrity of degenerative lumbar disks to prevent secondary diseases such as osteochondrosis, recurrent lumbar disk herniation, or segmental instability. In this phase-I/II study, NDplus is being investigated for its clinical applicability, safety, and efficacy in the repair of herniated, nucleotomized disks, and of adjacent degenerated disks, if present. To date, autologous disk chondrocytes have not been transplanted into degenerative disks without previous disk herniation. This is possible in the presented study by injection of autologous cells not only in the nucleotomized, but also in the adjacent degenerated disks. As such, this is the first study to investigate a therapeutic as well as a prophylactic approach to treat degenerative disks of the lumbar spine.

The objective of phase I was to develop a safety profile. This study further aims at developing and validating known and new biologic markers for the quality and clinical efficacy of the product as requested in the context of identity, purity, and potency characteristics of the medicinal/investigational product.

## Clinical material and methods

### Trial description

The NDisc study is a non-confirmatory, prospective, multi-center, unmasked, randomized study with two phases aimed at gathering preliminary clinical information on NDisc plus and NDisc basic used in the repair of herniated disks. The objectives of Phase I were to develop a safety profile and to evaluate feasibility of NDplus. This report presents data from Phase I up to 6 weeks after transplantation (Visit 4). Eligible patients underwent baseline assessments, sequestrectomy, and postoperative assessment of Visit 2. After this initial surgery, all patients were randomized with a 1:1 allocation ratio to either NDplus ADCT or NDbasic. Patients with a lumbar disk herniation were classified according to the presence (ADD) or absence (HD) of a degenerative disk at the adjacent level by the investigators of the center. At Visit 3, patients underwent pre-implantation assessments, transplantation and post-transplantation assessments. A follow-up was performed at Visit 4. The visit plan is shown in Table 1 in more detail.

**Table 1:** Time and event schedule until Visit 4. In phase I there is a close monitoring of safety parameters and additional MRI assessment

Visit	1	2a	2b	2c	2d	2e	2f	3a	3b	3c–3j	3k	3l	4
Events	Scree-ning	Baseline	SE	2 h p.s.	6 h p.s.	24 h p.s.	36 h p.s.	Examination	Implant	P.t.	48 h p.t.	21 d p.t.	Follow-up
Time windows		45 d p.sc.		2 h ± 30 min	6 h ± 30 min	24 h ± 2 h	36 ± 2 h	90 d ± 15 d		Directly + every 6 h ± 30 min	48 h ± 30 min	21 d ± 2d	42 d ± 7
Tests		Pre-operative	Intra-operative	2 h	6 h	24 h	36 h	Pre-implant	During implant	Post implant 6 h-intervals	48 h post implant	21 d post implant	Follow-up
Informed Consent	x												
History	x												
Demography	x												
Medical and surgical history		x											
Vital signs, height, weight		x						x					x
Work status	x	x						x				x	x
Tobacco use		x						x				x	x
Review in-and exclusion criteria	x		x						x				
MRI	x	x						x			x	x	x
Serological analysis	x		x					x					
Blood pregnancy test	x												
Safety laboratory tests			x	x	x	x	x	x		x	x	x	x
Adverse event		x	x	x	x	x	x	x	x	x	x	x	x
Concomitant medication		x	x	x	x	x	x	x	x	x	x	x	x

*D* day, *h* hours, *min* minutes, *p.s.*post-sequestrectomy, *p.sc.* post-screenig, *p.t.* post transplant, *SE* sequestrectomy

### Clinical investigation population

Twenty-four patients with a single-level lumbar herniated disk were recruited consecutively in three different centers. The N-Disc trial aimed to include patients with symptomatic lumbar disk herniation who failed adequate conservative or interventional treatment approaches in accordance with the guidelines of the German Society of Neurosurgery and the German Society of Orthopedics and Orthopedic Surgery. Additionally, an MRI determined disk herniation at the treatment level needed to correlate with the primary symptoms. To minimize confounding, patients with significant comorbidities were excluded from the study. Further inclusion criteria needed to be met: (1) no previous lumbar spine surgery; (2) age between 18 to 60 years; (3) proficient enough in the German language to understand the study; (4) no magnetic resonance imaging (MRI) documented associated lumbar disease such as lumbar spinal stenosis, spondylolisthesis, or fracture. If patients showed an extensive damage of the annulus fibrosus intraoperatively that may subsequently pose a significant greater risk of recurrence or non-containment of the injected material, they were excluded from the trial and randomization was not carried out. Written informed consent has been obtained from each patient.

### Assessments and laboratory values

Overall patient disposition and demographics, neurological, and functional status were documented. Serology of HIV, hepatitis, and Treponema pallidum were determined preoperatively. Laboratory values such as interleukine-6 (IL-6) and C-reactive protein (CRP) as safety parameters were evaluated. All laboratory values were classified as normal or abnormal according to the laboratories normal ranges. Systolic and diastolic blood pressure and pulse rate as vital signs were assessed. Adverse events (AE) and serious adverse events (SAE) were documented. The analysis of adverse events was focused on treatment emergent adverse events (TEAE). A TEAE or TESAE is any AE or SAE that occurs during or after implant. Frequency and percentage of TEAEs were summarized according to the primary system organ class and preferred term and tabulated by treatment group.

### Magnetic resonance imaging

Preoperative MRI of the lumbar spine was performed in a standardized fashion on a 1.5-Tesla MRI scanner. The protocols included T1- und T2-weighted turbo spin echo sequences in sagittal and transversal planes, a sagittal STIR sequence, a sagittal thin slice double echo volumetry sequence, and a sagittal spin echo T2 multi-echo relaxometry sequence, additionally. Numerical and comparative measurements in disk height, volumetry, and T2 relaxations times of index and adjacent disks and categorial evaluation such as degeneration scale, and the presence or absence of stenosis were determined by MRI. Osteochondrosis was graded by Modic changes [13]. The sequences were time optimized to improve patient’s acceptance and enable integrability of study protocols in the local MRI schedules. Furthermore, an external institution (MEDIRI—medical imaging research institute, Heidelberg, Germany) adapted the protocol to the local scanners to arrange image data comparability between the different study centers. Additionally, the institution trained local radiographers in standardized performance and engineered a software for central image processing and analysis. All images were read by an independent radiologist with high-level experience in spine imaging, blinded to patients and time of image acquisition.

### Trial organization, registration, and ethical aspects

The NDisc trial is an ongoing study of which Phase I safety results are presented here. Ethics approval was attained in Germany at the National Physician Board, “Landesärztekammer” Sachsen-Anhalt and in Austria at the committee of the Medical University Innsbruck. Furthermore, the clinical trial approval was obtained at the Paul-Ehrlich-Institute (Langen, Germany) and the Austrian Agency for Health and Food Safety (Vienna, Austria). The study complies with the World Medical Association Declaration of Helsinki Ethical Principles for Medical Research Involving Human Subjects, Good Clinical Practice (GCP), national pharmaceutical acts in the participating countries Austria and Germany, and European guidelines for the conduct of clinical trials with medicinal products for human use. The trial is initiated and sponsored by TETEC Ag (B|Braun Aesculap AG shared company, Reutlingen, Germany), which is responsible for management and registration (EudraCT No: 2010-023830-22, ID NCT01640457). CenTrial GmbH (Tuebingen, Germany) is responsible for clinical trial submission, independent clinical monitoring, and pharmacovigilance. Mediri GmbH (Heidelberg, Germany) as a core imaging lab is responsible for the development of MRI protocols, data storage, and analyses. Laboratory values are investigated centrally by Synlab Services GmbH (Synlab MVZ, Leinfelden-Echterdingen, Germany). Accovion GmbH (Eschborn, Germany) is responsible for data management, biometrics, and medial writing. Quality audits were performed routinely by the Ministry of Health.

### Investigational medicinal product

NDisc plus is used for ADCT. NDisc plus is an injectable, in situ polymerizing gel initially consisting of two separate components. Component A is a liquid matrix composed of the cell culture medium, modified albumin, and hyaluronic acid. Component A also contains autologous inter-vertebral disk cells dissolved in cell culture medium supplemented with human serum, chondroitin sulfate, insulin, BMP-2, and ascorbate. Component B is a solution containing bis thio-polyethylene glycol. Component A and B polymerize in situ using a dedicated application system (special dual-chamber syringe) to form the desired hydrogel. NDisc basic is used as control in the NDisc study. In NDisc basic component A is a liquid matrix composed of cell culture medium, modified albumin, and hyaluronic acid, the cell suspension is replaced by an aliquot of cell culture medium without additives. Component B is not modified [14].

### Sequestrectomy

Surgery was performed by two trial-designated surgeons under general endotracheal anesthesia with the assistance of an operating microscope while the patient was in a prone position. Depending on the location of disk herniation, the spinal canal harboring the sequestrated disk material was exposed either by a minimal interlaminar fenestration or a translaminar approach. If intradiscal material had to be removed, a limited nucleotomy was also performed [15]. In case of a lateral lumbar herniation, a lateral extraforaminal approach was performed [16]. Right after the extraction of the disk tissue, it was transferred into a sterile transport vial and provided to TETEC AG for the GMP compliant manufacturing of the investigational medicinal product NDisc plus.

### Transplantation

Transplantation was performed 90 days after sequestrectomy. NDplus or NDbasic was applied via an injection with a dual-needle technique directly at the intended site of action. NDplus or NDbasic is to be transplanted in a comfortable lateral or abdominal position. After the treatment level is localized and local anesthesia is applied, the puncture of the intervertebral disk was taken contra-laterally to the side of the disk surgery. An injection needle was placed in the center of the disk space under image guidance and the medicinal product was injected. In case of an ADD, the same procedure described above was conducted additionally at the adjacent, proximally located disk. Positioning of the needles and the mandarin took place simultaneously to minimize radiation exposure [14].

### Statistical analysis

This is a non-confirmatory study without pre-specified decision-making rules or hypotheses. Except laboratory values, all statistical analyses are descriptive and exploratory and there was no adjustment of significance levels for multiple testing or interim analyses. For laboratory values, the Kolmogorov-Smirnov test was used for testing normal distribution. The unpaired Student’s *t* test, Mann-Whitney U test and Fisher’s exact test were used to analyze differences in laboratory values as applicable. A *P* value <0.05 was considered statistically significant. For CRP, values below detection levels (<0.02 mg/dL) were set to 0.02 and for IL-6, values below detection levels (<2.0 pg/mL) were set to 2.0. All values were expressed as mean ± SD. All results were presented by treatment group (NDplus and NDbasic). All data were pooled across study centers for analysis and safety evaluations for all subgroups or categories were also performed by pooling ADD and HD patients. Figures were designed using GraphPad Prism (version 5.0 for Mac OS X, GraphPad Software, La Jolla California USA, www.graphpad.com).

## Results

**Fig. 1:**
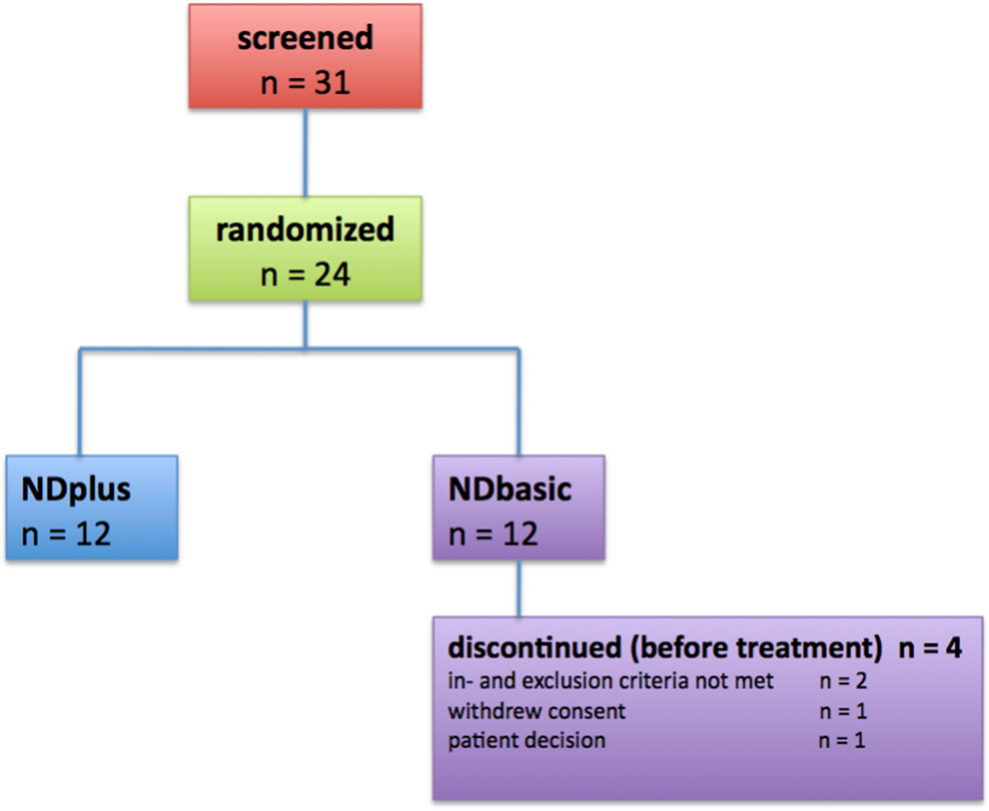
Twenty-four patients with single lumbar disc herniation were prospectively included in the study

The demographic details and preoperative characteristics of the patients are presented in Table 2. Twenty-four patients with single lumbar disk herniation were prospectively included in the study (Fig. 1). The proximally located disk was also degenerated in seven patients. The most commonly affected level of disk herniation was at L5/S1 in both groups. Twenty of the 24 patients were treated, 12 patients with the IMP NDisc plus (patients with ADD: two) and eight patients with the control preparation NDisc basic (patients with ADD: three). Four patients were excluded from the clinical trial before transplantation due to subsequently detected exclusion criteria, or, at their own request.

**Table 2:** Preoperative demographic and clinical characteristics

Characteristics		NDplus *n* = 12	NDbasic *n* = 12
Mean age in years (SD)		44.7 (6.7)	40.4 (9.8)
Sex	Female *n* (%)	2/12 (16.6)	8/12 (66.6)
	Male *n* (%)	10/12 (83.3)	4/12 (33.3)
Tobacco use	No *n* (%)	7/12 (58.3)	8/12 (66.6)
	Yes *n* (%)	5/12 (41.6)	4/12 (33.3)
Work status	Working full-time *n* (%)	10/12 (83.3)	9/12 (75.0)
	Working part-time *n* (%)	1/12 (8.3)	2/12 (16.6)
	Unemployed *n* (%)	1/12 (8.3)	0/12 (0.0)
	Not employed (e.g., homemaker, student) *n* (%)	0/12 (0.0)	1/12 (8.3)
	Retired *n* (%)	0/12 (0.0)	0/12 (0.0)
BMI (kg/m2)		24.2 (2.2)	24.9 (2.8)
Adjacent level degeneration	Presence of adjacent degenerative disc *n* (%)	2/12 (16.6)	5/12 (41.6)
	Absence of adjacent degenerative disc *n* (%)	10/12 (83.3)	7/12 (58.3)
Location of disc herniation	Between vertebra L3 and L4 *n* (%)	1/12 (8.3)	0/12 (0.0)
	Between vertebra L4 and L5 *n* (%)	3/12 (25.0)	3/12 (25.0)
	Between vertebra L5 and S1 *n* (%)	8/12 (66.6)	9/12 (75.0)
Prior analgesic medication	Subjects with prior analgesic medication	10/12 (83 %)	11/12 (91.7)
	Opioids, metamizole, paracetamol	5/12 (41.6)	6/12 (50)
	Non-steroidal anti-inflammatory drugs (e.g., diclofenac, naproxen)	6/12 (50.0)	11/12 (91.7)

*BMI* body mass index, *n* number of patients, *SD* standard deviation

The mean volume of injection was 1.19 mL.

Eight patients (*n* = 6 in the NDplus and *n* = 2 in the NDbasic group) experienced TEAEs, all of which were of mild or moderate intensity. Only two patients had TEAEs that were assessed by the investigator as related to the medical intervention or to either of the study treatment. One patient of the NDisc basic group experienced spinal pain 21 days post implant (non serious TEAE) assessed as related to both surgery and study treatment. One patient of the NDisc plus experienced an intevertebral disk protrusion (serious TEAE) assessed also as related to both surgery and study treatment. The patient underwent further surgery. No further spinal complications occurred in phase I. Considering the small number of patients with ADD, no differences were apparent between patients with and without ADD with respect to TEAEs and inflammatory parameters. The most commonly occurring TEAE was nasopharyngitis in three patients in the NDplus group and this was not related to the sequestrectomy or IMP.

**Fig. 2:**
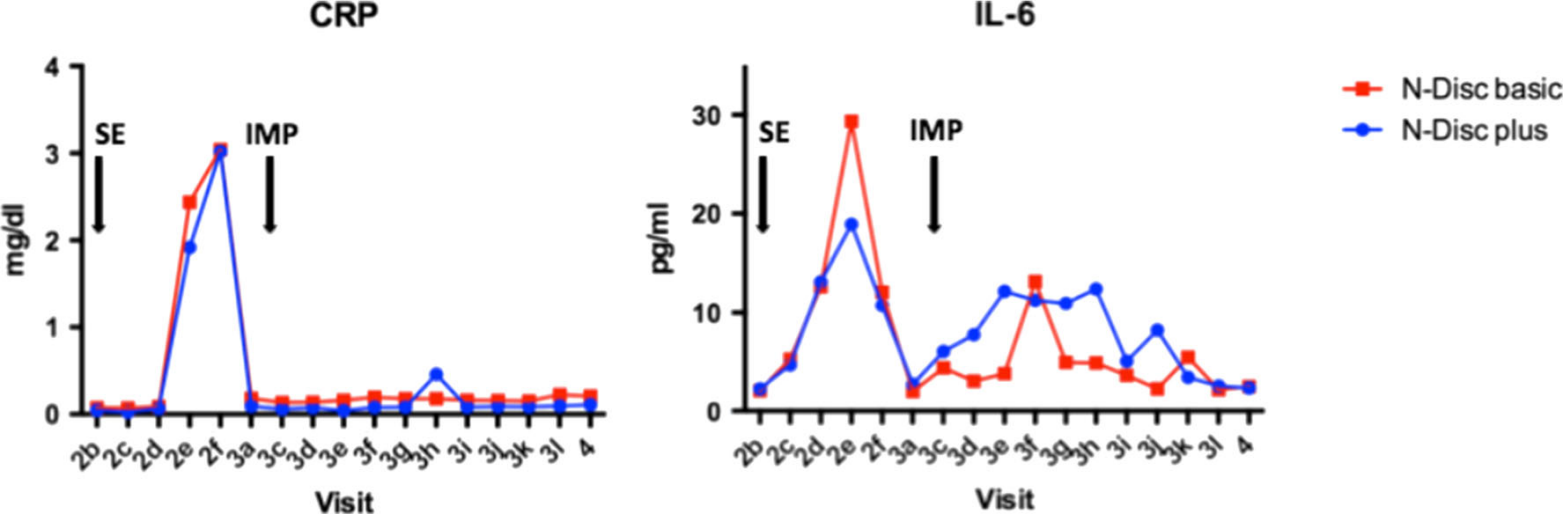
Laboratory values. *SE* sequestrectomy, *IMP* implantation

Laboratory parameters in both treatment groups increased temporarily after 36 h of sequestrectomy (CRP: NDplus group 3.0 ± 3 mg/dL vs. NDbasic group 3.0 ± 4 mg/dL; IL-6: NDplus group 10.7 ± 6 pg/mL and NDbasic group 12.0 ± 13 pg/mL) and turned to normal thereafter. CRP did not change after implantation, whereas IL-6 showed minor changes with a peak at 42 h post implantation. In the NDplus cohort IL-6 was elevated over a broader range, but this was not statistically significant (*p* > 0.05) (Fig. 2).

Post implantation there were no relevant changes in systolic and diastolic blood pressure and pulse rate from base line.

**Fig. 3:**
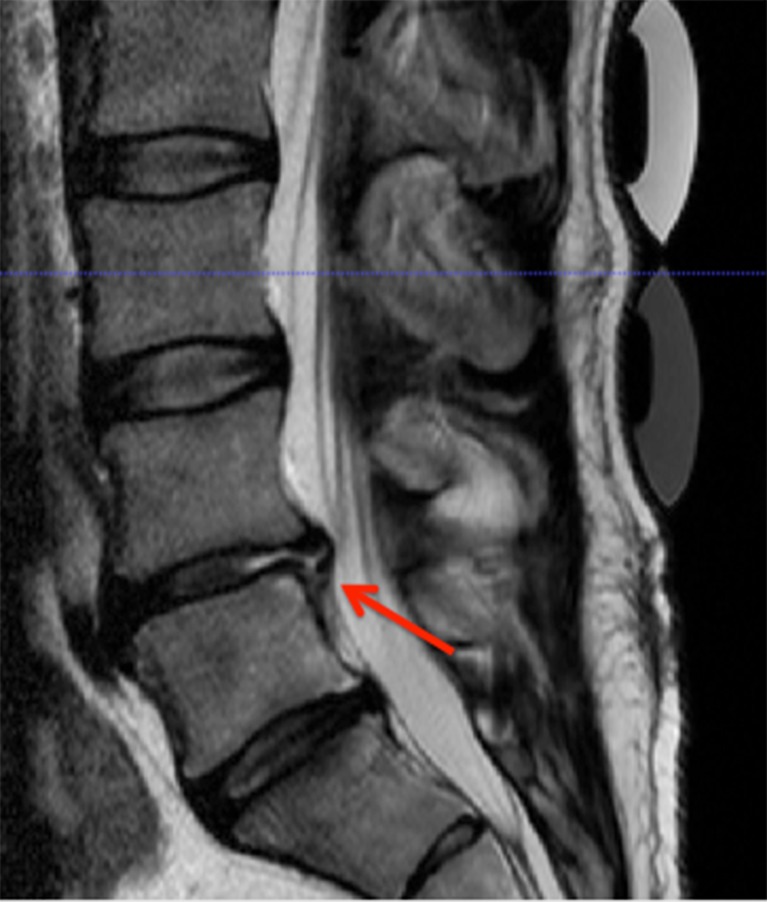
Post-transplant T2-weighted MRI. An extradiscal fluid collection (➔) was observed in three patients after transplantation surgery

In the MRI, extradiscal fluid collection (EDFC) was observed in three patients (*n* = 2/12 in the NDplus group vs. *n* = 1/8 in the NDbasic group) after the implantation, but did not have any space-occupying effect (see Fig. 3): in one patient in each group at 48 h and in two patients in the NDplus group at 21 days after transplantation. One of these patients demonstrated a recurrent disk herniation, which later also required surgery (7 months postoperative).

No fractures or more than moderate degenerative foraminal stenosis and degenerative spinal stenosis were observed. Osteochondrosis assessed by Modic grading of MRIs did not appear to worsen in any of the disk types during Phase I.

## Discussion

We have described the safety results of the first randomized prospective clinical study for ADCT in the treatment of surgically treated lumbar disks and in degenerated adjacent. The goal of ADCT is to reduce the degenerative sequel after lumbar disk surgery or to prophylactically avoid degeneration in adjacent disks. Injection of material in the disk, however, theoretically carries several risks such as extrusion of the injected material, increased intradiscal pressure potentially provoking disk herniations, and local or systemic inflammatory reactions.

Current treatment strategies for IVDD are aggressive surgical removal of the pathological disk or spondylodesis that do not address options of restoring structural or biological deteriorations of the IVD as the underlying problem. The rate of lumbar fusion surgery is rising dramatically compared to other musculoskeletal surgical procedures [17]. Patient self-rating for unsuccessful treatment after fusions for degenerative conditions ranges from 30 to 40 % [18]. Moreover, major complication rates of up to 20 % and long-term consequences of spinal fusion like adjacent segment degeneration of 37 % within 10 years have to be kept in mind [2]. Thus, lumbar fusion does not sufficiently improve the patient’s condition in many ways. Better outcomes were expected by substantially reducing the tissue damage with the introduction of minimal invasive spine surgery [3, 4]. Nevertheless, ADCT may offer a less invasive, biological alternative to lumbar fusion in an attempt to address LBP associated with IVDD earlier in the degenerative cascade.

Surgical interventions entail physiological responses including the rise of inflammatory cytokines [19]. Additionally, the reintroduction of disk cells to initiate cellular regeneration may also reactivate the innate immune system and potentially even specific immune responses. Therefore, observation of a pro-inflammatory response gives an early warning of potential complications like an autoimmune response against the implanted biomaterial. CRP is a long-term clinical standard to monitor innate responses and is independent of pain management strategies [19]. Among other pro-inflammatory cytokines, IL-6 may be regarded as an important component contributing to the local inflammatory process in disk herniations [20]. Moreover, serum levels of IL-6 may be increased in patients with lumbar radicular pain due to disk herniation [21]. There is no data in the literature, which role serum IL-6 plays after lumbar sequestrectomy. In our study population, CRP and IL-6 were evaluated 36 h after surgery. Furthermore, IL-6 increased 42 h after implantation, but turned to normal values thereafter. Thus, there is no indication of relevant immunological consequences of the intradiscal injection at short-term follow-up, neither in the NDisc plus nor in the NDisc basic cohort.

Routine treatment (elective sequestrectomy) in the target patient population was considered to be associated with AEs such as recurrent disk herniation or ongoing or recurrent low back pain or sciatica in up to 25 % of patients within 2 years. Symptomatic reherniations occur in approximately 10 % of patients with the highest risk within the first 6 months [15]. Recurrent symptoms due to disk degeneration or osteochondrosis also termed post-discectomy syndrome may occur over time [22]. In the present Phase I study, there was one reherniation requiring reoperation early after sequestrectomy and before implantation was scheduled, which is clearly unrelated to the injection substrate. Thus, no indication for an increased risk of reherniations due to the injected biomaterial was observed.

Early postoperative routine MRI is difficult to interpret due to postoperative changes. It is well known, that (residual) intervertebral disk protrusions are common on MRI after sequestrectomy [23]. It is therefore questionable if MRI in the early postoperative period after lumbar surgery is meaningful. Early postoperative MRI must be interpreted with caution, since correlation between clinical and radiological findings is weak [24]. The MRI data in Phase I revealed a minimal EDFC in three patients. This may also occur after routine disk surgery. As previously described, in one case of EDFC, a patient developed a disk reherniation after transplantation, which required surgery 7 months after the index operation. Nonetheless, given EDFC as a common finding after disk surgery it is doubtful that the finding of small EDFC represents an elevated risk of reherniation.

### Overall conclusions

Overall, the rates of radiological and clinical reherniations as well as of AEs in Phase I of this trial are comparable with those expected in the early time course after elective disk surgery. No indications of harmful material extrusion or immunological consequences due to the IMP NDplus were observed. Therefore, the study appears to be safe and feasible. Safety analyses of Phase I of this trial indicate a relatively low risk considering the benefits that patients with debilitating degenerative disk disease may gain. Regardless of the promising results of this Phase I study, further analyses are necessary and warranted.
